# Hypoxia-inducing factors as master regulators of stemness properties and altered metabolism of cancer- and metastasis-initiating cells

**DOI:** 10.1111/jcmm.12004

**Published:** 2013-01-10

**Authors:** Murielle Mimeault, Surinder K Batra

**Affiliations:** Department of Biochemistry and Molecular Biology, College of Medicine, Eppley Cancer Institute, University of Nebraska Medical CenterOmaha, NE, USA

**Keywords:** Hypoxia, Hypoxia-inducible factors, Metabolic pathways, Cancer progression, Metastases, Cancer stem/progenitor cells, Cancer-initiating cells, Metastasis-initiating cells, Targeted therapies

## Abstract

Accumulating lines of experimental evidence have revealed that hypoxia-inducible factors, HIF-1α and HIF-2α, are key regulators of the adaptation of cancer- and metastasis-initiating cells and their differentiated progenies to oxygen and nutrient deprivation during cancer progression under normoxic and hypoxic conditions. Particularly, the sustained stimulation of epidermal growth factor receptor (EGFR), insulin-like growth factor-1 receptor (IGF-1R), stem cell factor (SCF) receptor KIT, transforming growth factor-β receptors (TGF-βRs) and Notch and their downstream signalling elements such as phosphatidylinositol 3′-kinase (PI3K)/Akt/molecular target of rapamycin (mTOR) may lead to an enhanced activity of HIFs. Moreover, the up-regulation of HIFs in cancer cells may also occur in the hypoxic intratumoral regions formed within primary and secondary neoplasms as well as in leukaemic cells and metastatic prostate and breast cancer cells homing in the hypoxic endosteal niche of bone marrow. The activated HIFs may induce the expression of numerous gene products such as induced pluripotency-associated transcription factors (Oct-3/4, Nanog and Sox-2), glycolysis- and epithelial-mesenchymal transition (EMT) programme-associated molecules, including CXC chemokine receptor 4 (CXCR4), snail and twist, microRNAs and angiogenic factors such as vascular endothelial growth factor (VEGF). These gene products in turn can play critical roles for high self-renewal ability, survival, altered energy metabolism, invasion and metastases of cancer cells, angiogenic switch and treatment resistance. Consequently, the targeting of HIF signalling network and altered metabolic pathways represents new promising strategies to eradicate the total mass of cancer cells and improve the efficacy of current therapies against aggressive and metastatic cancers and prevent disease relapse.

- Introduction
- Critical functions of HIFs in the acquisition of more malignant phenotypes and behaviour by cancer- and metastasis-initiating cells and their differentiated progenies
- Functions of HIF-1a in leukaemic stem/progenitor cells and their differentiated progenies and novel targeted therapies
- Functions of hypoxia and HIFs in the development of glioblastoma multiforme and targeted therapies
- Functions of hypoxia and HIFs in the development of melanoma and targeted therapies
- Functions of hypoxia and HIFs in the development of prostate cancer and metastases
  – Novel therapies by targeting HIFs and altered metabolic pathways in PC stem/progenitor cells and their differentiated progenies
- Functions of hypoxia and HIFs in the development of breast cancer and metastases
  – Molecular targeting of HIFs and altered metabolic pathways in BCSCs and their differentiated progenies
- Functions of hypoxia and HIFs in the development of pancreatic cancer and metastases
  – Novel therapies by targeting HIFs and altered metabolic pathways in pancreatic stem/progenitor cells and their differentiated progenies
- Conclusions and perspectives

## Introduction

**Fig. 1:**
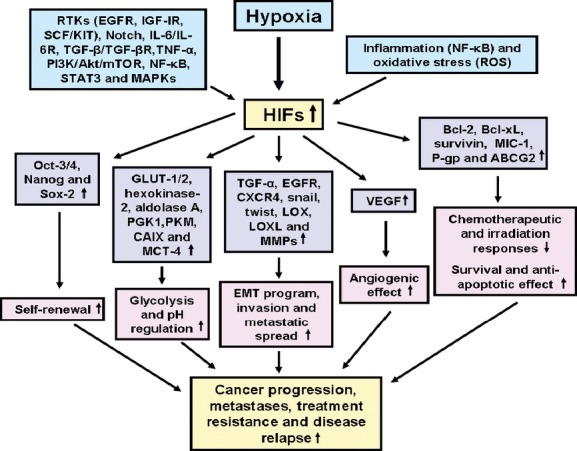
Cellular events and signalling elements involved in the regulation of the stabilization and activation of hypoxia-inducible factors. The increase in the stability and activation of HIFs, HIF-1α and HIF-2α, in cancer cells including cancer stem/progenitor cells, which may be induced *via* different growth factor and cytokine pathways under normoxic and hypoxic conditions, hypoxic microenvironment and inflammation are illustrated. The potential cellular signalling elements modulated through the up-regulation of HIFs and which can contribute to high self-renewal, altered glycolytic metabolism, invasion, metastases, treatment resistance and disease relapse are also indicated. BCRP/ABCG2: breast cancer resistance protein; CAIX: carbonic anhydrase; EGFR: epidermal growth factor receptor; GLUT: glucose transporter; IL-6: interleukin-6; MAPK: mitogen-activated protein kinase; MCT-4: monocarboxylate transporter-4; MIC-1: macrophage inhibitory cytokine-1; MMPs: metalloproteinases; mTOR: molecular target of rapamycin; NF-κB: nuclear factor-κB; RTK: receptor tyrosine kinase; PI3K: phosphatidylinositol 3′-kinase; PGK1: phosphoglycerate kinase 1; PKM: pyruvate kinase M; P-gp: P-glycoprotein; ROS: reactive oxygen species; TGF-β: transforming growth factor-β; TNF-α: tumour necrosis factor-α; STAT3: signal transducer and activator of transcription 3; VEGF: vascular endothelial growth factor.

Recent advances in cancer research have indicated that the enhanced expression and activation of hypoxia-inducible factors (HIFs) frequently occur in cancer cells during cancer progression and is associated with their acquisition of a more malignant behaviour, treatment resistance and poor outcome of cancer patients (Fig. 1) [1–12]. Particularly, responses of cancer cells to environmental stress as observed for their normal counterparts generally implicate the induction of HIFs [1, 3–5, 9–16]. HIF transcription factors of the basic helix-loop-helix (bHLH)/PAS family include HIF-1α that is differently expressed in most tissues and HIF-2α which shows a more restricted tissue expression pattern in various locations, including kidneys, brain, lungs, liver, gastrointestinal tract, pancreas and heart [1, 2, 17–20]. The regulation of the cellular stability and activity of HIF-1α and HIF-2α in normal cells and cancer cells is highly dependent on oxygen supply (Figs 4) [3, 18, 21, 22]. In fact, oxygen-sensitive HIF-α proteins are generally hydroxylated by prolyl hydroxylase domain (PHD) proteins under normoxic conditions [21, 23]. This molecular event results in the interaction of HIF-α with Von Hippel-Lindau (VHL) tumour suppressor gene product, which is a component of the protein complex possessing a ubiquitin ligase E3 activity, that targets the HIF-α subunit for polyubiquitylation and subsequent proteasomal degradation (Fig. 3) [22, 23]. In contrast, the HIF-α subunit does not interact with the VHL protein in the presence of low oxygen levels and translocates to the nucleus where it forms a heterodimer with the constitutively expressed HIF-β partner subunit, also designated as aryl hydrocarbon receptor nuclear translocator (ARNT) that specifically binds to hypoxia-responsive elements (HREs) found in target gene promoters (Fig. 3) [3, 24].

**Fig. 2:**
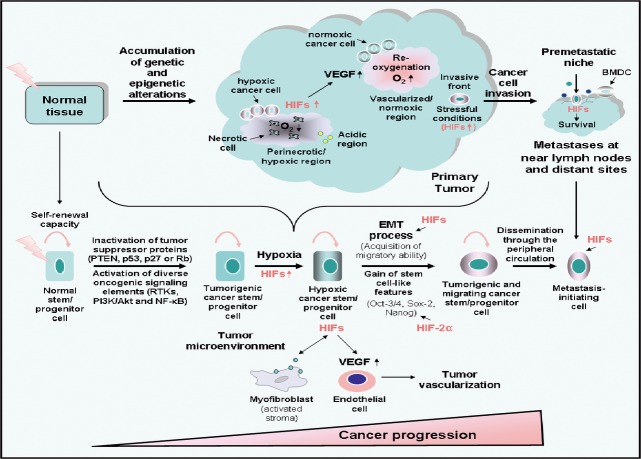
Proposed model of malignant transforming events associated with cancer progression and metastases driving hypoxia and enhanced expression of HIFs in cancer stem/progenitor cells. Genetic and epigenetic alterations occurring in tissue-resident adult stem/progenitor cells during intense injury, oxidative stress, inflammation and/or ageing may result in their malignant transformation into tumourigenic cancer stem/progenitor cells also designated cancer-initiating cells that are able to generate the bulk mass of heterogeneous and differentiated cancer cells within tumour. The scheme also shows the potential localization of clusters of cancer stem/progenitor cells expressing HIFs at the hypoxic region near a necrotic areas and invasive front of the primary tumour. The enhanced expression of HIFs in highly tumourigenic cancer stem/progenitor cells and their differentiated progenies, which may be induced under hypoxia or sustained activation of growth factor pathways and PI3K/Akt/mTOR under normoxic and hypoxic conditions, may promote the EMT programme, altered metabolic pathways and re-expression of stem cell-like markers such as Oct-3/4, Sox-2 and Nanog and pro-angiogenic factor VEGF. These molecular transforming events in turn may contribute to the acquisition of a more malignant behaviour and migratory ability by cancer cells and tumour neovascularization. Moreover, bi-directional cross-talks between cancer cells and stromal myofibroblasts found within their local tumour microenvironment also may promote their gain of more aggressive phenotypes. Hence, highly tumourigenic and migratory cancer stem/progenitor cells with stem cell-like properties that survive under stressful conditions, including low oxygen tension and nutrient deprivation, during primary cancer progression and reach the invasive front of primary tumour can be involved in dissemination and metastatic spread at near lymph nodes and distant tissues. The disseminated cancer stem/progenitor cells that are able to survive in their novel microenvironment prevalent at metastatic sites can give rise to the total mass of differentiated cancer cells forming secondary tumours. The preferential migration of cancer cells to pre-metastatic niches induced by different soluble factors released from primary tumour and bone marrow-derived cells (BMDCs) at distant sites is also illustrated.

**Fig. 3:**
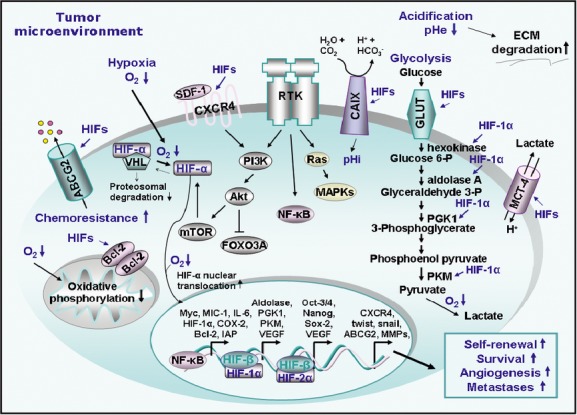
Scheme showing the potential molecular events induced in cancer cells in the hypoxic tumour microenvironment. The intracellular consequences of decreased oxygen level (hypoxia) in cancer cells including the switch of mitochondrial oxidative phosphorylation to anaerobic glycolysis and enhanced nuclear translocation of HIF-α subunit are illustrated. The enhanced stabilization and activation of HIF-1α and HIF-2α and their formation of nuclear heterodimers with HIF-β receptor in cancer cells under hypoxia that in turn may result in the transcriptional activation of numerous gene products involved in anaerobic glycolysis, pH regulation, self-renewal, survival and induction of angiogenic switch and metastases are indicated. The enhanced cellular accumulation and activation of HIF-α protein subunit which may be induced through the stimulation of different receptor tyrosine kinases (RTKs) in cancer cells under normoxic and hypoxic conditions are also illustrated. Particularly, the stimulation of RTKs may lead to the sustained activation of phosphatidylinositol 3′-kinase (PI3K)/Akt/molecular target of rapamycin (mTOR) pathway that in turn may induce the translational machinery and HIF protein synthesis and/or enhanced stabilization of HIF-α subunit. Moreover, the activation of RTKs may result in the stimulation of nuclear factor-kappaB (NF-κB) that in turn can induce the transcriptional up-regulation of HIFs. ABCG2/BCRP: breast cancer resistance protein; CAIX: carbonic anhydrase IX; COX-2: clyooxygenase-2; ECM: extracellular matrix; FOXO3A: forehead 3A; GLUT: glucose transporter; HIFs: hypoxia-inducible factors; IAP: inhibitor of apoptosis protein; IL-6: interleukin-6; MAPK: mitogen-activated protein kinase; MCT: monocarboxylate transporter; MIC-1: macrophage inhibitory cytokine-1; MMPs: matrix metalloproteinases; pHe: extracellular pH; pHi: intracellular pH; PGK1: phosphoglycerate kinase 1; PKM: pyruvate kinase M; VEGF: vascular endothelial growth factor.

**Fig. 4:**
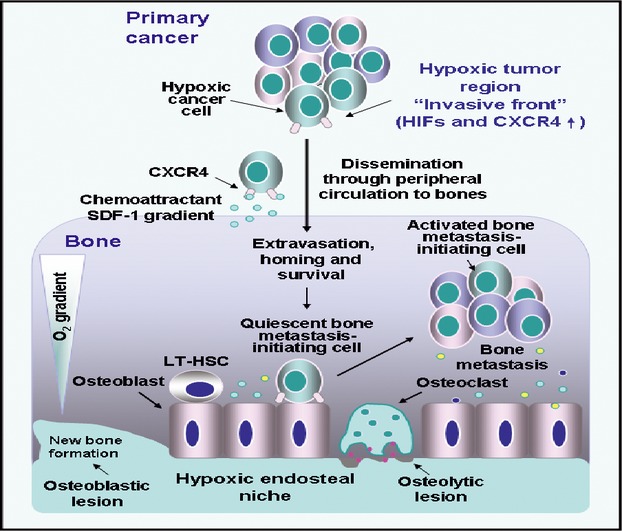
Proposed model of potential transforming events occurring in hypoxic cancer cells during epithelial cancer progression and bone metastasis. The up-regulated expression levels of stem cell-like phenotypes, HIFs, CXC chemokine receptor (CXCR4) and occurrence of the EMT programme in prostate or breast cancer cells within the hypoxic region at the invasive front of the primary tumour may lead to their invasion and dissemination through the peripheral circulation and homing at distant metastatic sites. More specifically, circulating prostate or breast cancer cells expressing high level of CXCR4 can preferentially disseminate and home to specific metastatic sites such as bones at least in part through the chemoattractant gradient formed by stromal cell-derived factor-1 (SDF-1) released by endothelial cells. The hypoxia-adapted prostate or breast cancer cells may compete with long-term haematopoietic stem cells (LT-HSCs) to occupy the hypoxic endosteal niche within BM and survive under a dormant state for a short or long period of time. The activation of dormant prostate or breast cancer cells may occur through the release of different growth factors and cytokines by cancer cells and stromal host cells under specific microenvironmental conditions. The activated prostate or breast cancer cells can give rise to the total tumour cell mass and skeletal metastasis formation. The bone metastases of prostate cancer cells are predominantly associated with the formation of osteoblastic lesions (bone formation), whereas bone metastases of breast cancer cells are generally related with the formation of osteolytic lesions (bone destruction).

In addition to the up-regulation of HIF activity under low oxygen tension, the stability and activation of HIF-1α and HIF-2α in cancer cells may also be differently regulated through the sustained stimulation of different growth factor and cytokine pathways and oxidative stress under normoxic and hypoxic conditions (Figs 1 and 3) [25–33]. The growth factor signalling elements include different receptor tyrosine kinases (RTKs), such as epidermal growth factor receptor (EGFR/erbB1), HER2/erbB2/Neu, insulin-like growth factor-1 receptor (IGF-1R), stem cell factor (SCF)/KIT receptor, Notch, interleukin-6/IL-6R receptor and transforming growth factor-β/TGF-βR receptors [25–39]. These tumourigenic pathways may cooperate to stimulate different downstream signalling elements, including phosphatidylinositol 3′-kinase (PI3K)/Akt/molecular target of rapamycin (mTOR), Ras/mitogen-activated kinase (MEK)/extracellular signal-regulated kinase (ERK), nuclear factor-kappaB (NF-κB) and signal transducer and activator of transcription 3 (STAT3) pathways that in turn up-regulate the expression and/or stability of HIF-α subunit [25–40]. Also, inactivation of tumour suppressor proteins such as pVHL, phosphatase tensin deleted on chromosome 10 (PTEN) and p53 in cancer cells may impair HIF degradation and/or enhanced PI3K/Akt activation and lead to HIF-α protein accumulation and increased expression of many HIF-regulated genes under normoxic and hypoxic conditions [1, 22, 28, 38, 41, 42]. Hence, HIF transcription factors can activate the transcription of numerous gene products that contribute to the malignant reprogramming of cancer cells during cancer progression and metastases and tumour angiogenic switch (Figs 1 and 3) [4, 25–33, 36, 37, 39, 41–44].

Importantly, recent findings have also indicated that HIF-1α and HIF-2α play critical roles for the gain of more malignant phenotypes by highly tumourigenic cancer stem/progenitor cells endowed with stem cell-like properties also designated as cancer-, tumour-, and metastasis-initiating cells that are able to generate the bulk mass of heterogeneous and differentiated cancer cells within tumours and which are involved in primary cancer progression, metastases, resistance to current cancer therapies and disease relapse [14, 45–53]. Thus, the complete eradication of the total mass of tumour cells, including hypoxic and normoxic cancer stem/progenitor cells and their differentiated progenies, by targeting the HIF signalling network might be crucial to improve current cancer therapies and prevent disease relapse. New therapeutic strategies such as guanine-rich oligodeoxynucleotides (G-rich ODNs), chemical compounds and chemotherapeutic drugs that target HIF-1α and/or HIF-2α proteins and repress the expression of their target genes in various cancer cells have been shown to counteract primary cancer progression and metastases at distant sites, and reverse treatment resistance under normoxic and hypoxic conditions (Tables 1 and 2) [11, 39, 44, 54–66]. In this matter, we review recent findings indicating pivotal roles of HIF-1α and HIF-2α in the modulation of stemness properties and altered metabolic pathways of cancer stem/progenitor cells and their differentiated progenies during the development of diverse aggressive and recurrent cancers. The emphasis is on the functions of HIFs during leukaemogenesis and the development of glioblastoma multiforme, melanoma and prostate, breast and pancreatic cancers and novel targeted therapies directed against HIFs and altered metabolic pathways, including glycolysis, lipogenesis and autophagy.

**Table 1:** Potential therapeutic strategies targeting altered growth factor and intracellular elements in normoxic and hypoxic cancer- and metastasis-initiating cells and their differentiated progenies

Molecular target	Name of inhibitory agent
Hypoxia-activated pro-drug	
Hypoxic cancer cells	TH-302
Growth factor signalling elements	
EGFR (erbB1)	Anti-EGFR mAb (cetuximab or IMC-C225 (Erbitux®)*, panitumumab (Vectibix®)*, mAb-C225); TKI (gefitinib (Iressa®)*, erlotinib (Tarceva®)*, AG1478)
HER-2 (erbB2)	Anti-HER-2 mAb (trastuzumab (Herceptin®)*)
IGF-1R	Anti-IGF-1R mAb (robatumumab or R1507); TKI (NVP-AEW541, BMS-536924)
TGF-βRI	SD-208
KIT	Imatinib mesylate (Gleevec®)*, dasatinib (Sprycel®)*nilotinib or AMN107 (Tasigna®)*, bafetinib (INNO-406)
Notch	γ-secretase inhibitor (DAPT, MK-0752, GSI-18)
Nodal/Cripto	Anti-Cripto mAb, LEFTY
Wnt/β-catenin	Anti-Wnt antibody, AV65, WIF-1
CXCR4	Anti-CXCR4 mAb, CXCR4 antagonist (plerixafor or AMD3100 (Mozobil®)*)
VEGF	Anti-VEGF mAb (bevacizumab (Avastin®)*)
VEGFR-2	Anti-VEGFR-2 mAb (ramucirumab or IMC-1121B, DC101 or IMC-1C11)
VEGFR-2, EGFR, RET-TKI	MKI (Vandetanib or ZD6474)
VEGFRs, PDGFRs, KIT	MKI [Sunitinib (Sutent®)*, Axitinib or AG-013736 (Inlyta®)*]
VEGFR-2 and -3, PDGFRs, KIT, B-Raf, C-Raf	MKI (Sorafenib (Nexavar®)*)
VEGFRs, PDGFRs, FGFR-1, FGFR-3, KIT, Itk, Lck and c-Fms	MKI (Pazopanib (Votrient®)*)
Intracellular signalling elements	
HIFs	YC-1, P276-00, P3155, NSC-134754, PX-478, KCN1 G-rich ODNs (JG243 and JG244), anthracyclins, 2-methoxyestradiol, echinomycin, Rakicidin A
PI3K	LY294002, Wortmannin
mTOR	Everolimus or RAD001 (Afinitor, Zortress or Certican®)*, temsirolimus or CCI-779 (Torisel®)*, sirolimus or rapamycin (Rapamune®)*
PI3K/mTOR	PI-103, NVP-BEZ235
Ras	*S*-*trans trans*-farnesylthiosalicylic acid
NF-κB	bortezomib (Velcade®)*, sulphasalazine (Azulfidine®)*, salinosporamides A (NPI-0052), parthenolide IkBα inhibitor (PS-341)
STAT3	G-rich ODN (T40214)
COX-2	Etodolax (Lodine SR or Eccoxolac®)*, NS-396
Bcl-2 and/or Bcl-xL	AS (oblimersen sodium (Genasense®)*), ABT-263
BCR-ABL	Imatinib mesylate (Gleevec®)*, dasatinib (Sprycel®)* nilotinib or AMN107 (Tasigna®)*, bafetinib (INNO-406)

* Trade name of Food and Drug Administration (FDA) approved drug for treating specific cancer(s) and/or other disorder(s).

c-Fms: transmembrane glycoprotein receptor tyrosine kinase; AS: antisense oligonucleotide; COX-2: clyooxygenase-2; CXCR4: CXC chemokine receptor 4; DAPT: *N*-(N-3,5-difluorophenacetyl)-L-alanyl]-S-phenylglycine t-butyl ester; EGFR: epidermal growth factor receptor; G-rich ODNs: guanine-rich oligodeoxynucleotides; FGFR: fibroblast growth factor receptor; IGF-RI: insulin-like growth factor-1 receptor; Itk: interleukin-2 receptor inducible T-cell kinase; KCN1: 3,4-dimethoxy-N-[(2,2-dimethyl-2H-chromen-6-yl)methyl]-N-phenylbenzenesulpfonamide; Lek: leucocyte-specific protein tyrosine kinase; mAb: monoclonal antibody; MKI: multi-targeted kinase inhibitor; NF-κB: nuclear factor-kappaB; PI3K: phosphatidylinositol 3′-kinase; STAT3: signal transducer and activator of transcription 3; TGF-βR: transforming growth factor-β receptor; TKI: tyrosine kinase inhibitor; VEGF: vascular growth factor; Wnt: Wingless ligand.

**Table 2:** Potential therapeutic strategies targeting altered metabolic and pH regulatory signalling pathways in normoxic and hypoxic cancer- and metastasis-initiating cells and their differentiated progenies

Molecular therapeutic target	Name of inhibitory agent
Altered metabolic signalling elements	
Glucose transporters	2-deoxy-D-glucose
Hexokinase-2	Lonidamine
Glyoxalase-1	*S*-*p*-bromobenzylglutathione
FASN	Orlistat, cerulenin, C75, resveratrol
Monoacylglycerol lipase	JZL184
Autophagy	Bafilomycin A1, 3-methyladenine, chloroquine (Aralen®)*, hydrochloroquine (Plaquenil®)*
pH regulatory signalling element	
CAIX	Anti-CAIX mAbs (M75 and G250), glycosyl coumarins (GC-204 and GC-205), sulphonamides (CAI17, ureido-sulphonamide, U-104)

* Trade name of Food and Drug Administration (FDA) approved drug.

CAIX: carbonic anhydrase IX; FASN: fatty acid synthase.

## Critical functions of HIFs in the acquisition of more malignant phenotypes and behaviour by cancer- and metastasis-initiating cells and their differentiated progenies

A growing body of experimental evidence has indicated that most human cancers may originate from the accumulation of different genetic and epigenetic alterations in tissue-resident adult stem cells and/or their early progenitors with stem cell-like properties during the lifespan resulting in their malignant transformation into cancer stem/progenitor cells [45, 47, 49, 52, 53, 67–73]. In support of this, cancer cells expressing stem cell-like markers and endowed with a high self-renewal ability and aberrant differentiation potential have been identified and isolated from primary neoplasms, peripheral circulation and metastatic tissue specimens [45, 53, 68, 71, 73–83]. Cancer types harbouring a cancer stem/progenitor cell subpopulation include leukaemias, brain tumours, melanomas and various epithelial cancers such as lung, liver, gastrointestinal, colorectal, pancreatic, breast, ovarian and prostate cancers [45, 47, 53, 68, 71, 73–77, 79, 80, 82–86]. It has been shown that cancer stem/progenitor cells can give rise to the total mass of heterogeneous and differentiated cancer cells within primary and secondary tumours and play key roles for invasion, metastases at regional and distant tissues as well as tumour angiogenesis [45, 47, 49, 52, 53, 68, 69, 71, 73–77, 79, 82, 83, 87–89]. New findings have also indicated that cancer- and metastasis-initiating cells with stem cell-like features typically display a higher resistance than the bulk mass of differentiated cancer cells to oxygen and nutrient deprivation and current anti-hormonal, radiation therapy and chemotherapy, and thereby they can be responsible for disease relapse [6, 12, 49, 51–53, 56, 73–77, 79, 82, 83, 88, 90–96].

Recently, the cancer stem cell hypothesis has also been reviewed to consider the complexity of molecular transforming events that may occur in these immature cancer cells and their differentiated progenies as well as their local microenvironment under normoxic and hypoxic conditions during cancer development [6, 47, 49, 82, 83]. Based on the cancer stem cell concept, cancer heterogeneity may be attributed to the differences between tissue-resident stem/progenitor cell types at the origin of particular cancer subtypes as well as progressive changes in the number and phenotypes of these immature cancer cells and their differentiated progenies and their local microenvironment during disease progression and after treatment initiation (Fig. 2) [7, 47, 82, 83, 97]. In this regard, rapidly growing tumours are typically characterized by disorganized vasculature with an abnormal leaky and tortuous structure [5, 6, 7, 98–100]. These rapidly growing tumours also exhibit hypoxic intratumoral regions that did not supply sufficient oxygen and nutrients to cells and require a high adaptation of cancer cells for their survival [5–7, 98–100]. It has been shown that changes in the local microenvironment of cancer stem/progenitor cells and their progenies, including the induction of hypoxic intratumoral regions within poorly vascularized tumours, may result in alterations of different gene products that contribute to their acquisition of more aggressive phenotypes and survival advantages [1, 7, 14, 46, 48, 50, 51, 66, 89, 98–104]. More specifically, it has been observed that hypoxia and enhanced expression and activity of HIF-1α and HIF-2α in cancer stem/progenitor cells and their progenies frequently occur during disease progression and metastases and may result in the up-regulation of different stemness gene products and survival signalling elements [6, 14, 48, 50, 90, 94, 99, 102, 104, 105]. The altered gene products modulated by HIFs comprise the regulators of induced pluripotency (octamer-binding transcription factor Oct-3/4, Sox-2 and Nanog), epithelial-mesenchymal transition (EMT) programme (EGFR, CXCR4, snail and twist), glucose transporters (GLUT-1/2) involved in the glucose uptake, altered metabolic pathways such as glycolytic enzymes, microRNAs (miRNAs) and drug resistance-associated molecules (ABCB2, Bcl-2, Bcl-xL, survivin and macrophage inhibitory cytokine-1 ‘MIC-1’; Figs 3) [4, 14, 22, 25, 51, 99, 100, 106–110]. These HIF-induced signalling elements may provide critical functions for high self-renewal capacity, energy supply through enhanced aerobic and anaerobic glycolysis, invasion, metastases and treatment resistance of cancer- and metastasis-initiating cells and their differentiated progenies [4, 14, 22, 25, 99, 100, 106–112].

In addition, among other important gene products modulated by HIF-1α, monocarboxylate transporter-4 (MCT-4) and carbonic anhydrase IX (CAIX) are often overexpressed in several cancer types as compared with normal tissues and involved in the pH regulatory system of hypoxic cancer cells characterized by a high rate of glycolytic metabolism and production of acidic metabolites such as lactic and carbonic acids (Fig. 3) [113–116]. More specifically, MCT-4 can contribute to the export of lactate into extracellular space of cancer cells [115, 116]. Furthermore, CAIX is a dimeric transmembrane metalloenzyme that catalyses the rapid interconversion of carbon dioxide (CO_2_) molecules to bicarbonate (HCO_3_^−^) and protons (H^+^) in the extracellular space of hypoxic cancer cells [113–115]. Hence, bicarbonate generated by CAIX may be subsequently transported by different bicarbonate transporters such as sodium bicarbonate co-transporters into cancer cells, and thereby plays key roles for the maintenance of intracellular pH (pHi) necessary for the survival and growth of cancer cells [113–115]. Also, the generation of protons through the hydration of carbon dioxide by CAIX and accumulation of acidic metabolites in the local microenvironment of cancer cells may contribute to the acidification of extracellular pH (pHe) that favours the normal cell death, degradation of extracellular matrix (ECM), stromal invasion and metastases of cancer cells (Fig. 3) [113–115]. In addition, the induction of tumour angiogenesis by HIFs through the up-regulated expression of pro-angiogenic factors such as vascular endothelial growth factor (VEGF) in cancer stem/progenitor cells and their differentiated progenies may also promote the tumour growth and formation of invasive and metastatic cancers (Figs 3) [25, 35, 39, 106, 117–119].

Although a highly structural homology exists between HIF-1α and HIF-2α, these transcription factors may also be differently regulated and transactivate common and unique targeted gene products in a cancer cell-dependent manner under normoxic and hypoxic conditions (Fig. 3) [16, 20, 24, 89, 106, 107, 117, 120, 121–125]. Particularly, the differences between the tissue expression pattern and transactivation domains of HIF-1α and HIF-2α proteins and their recruitment of different transcriptional factors acting co-activator or co-repressor may lead to their activation of specific gene subsets in normal and cancer cells [20, 23, 24, 106, 122, 123]. The transcription factors that can cooperate with HIFs include different histone acetyl transferase (HAT) co-activators such as CREB-binding protein (CBP)/adenovirus E1A-binding protein p300 (p300) and proteins in steroid receptor co-activators (SRCs)/p160 family such as SRC-1 [20, 23, 24, 106, 122, 123]. In general, HIF-1α may specifically induce the enhanced expression of CAIX, lysyl oxidase (LOX) and glycolytic enzymes such as hexokinase-2, aldolase A, phosphoglycerate kinase 1 (PGK1) and pyruvate kinase M (PKM), whereas HIF-2α appears to preferentially up-regulate the gene products including TGF-α, Myc, cyclin D1 and embryonic stem cell-like markers such as Oct-3/4, Sox-2 and/or Nanog in cancer cells under normoxic and hypoxic conditions (Figs 1 and 3) [16, 106, 107, 117, 120, 121, 124–127]. In this regard, we review the molecular mechanisms controlling the expression levels and specific functions of HIF-1α and HIF-2α and their downstream signalling elements during initiation and progression of leukaemias, glioblastoma multiforme, melanoma and epithelial cancers such as prostate, breast and pancreatic cancers.

## Functions of HIF-1α in leukaemic stem/progenitor cells and their differentiated progenies and novel targeted therapies

Numerous investigations have revealed that leukaemias may originate from the accumulation of genetic and/or epigenetic alterations occurring in haematopoietic stem cells (HSCs), precancerous-LSCs (pre-LSCs) or more committed myeloid progenitor cells endowed with a high self-renewal ability and aberrant differentiation potential [45, 68, 69, 71, 87]. Importantly, recent lines of experimental evidence have also indicated that LSCs and their early progenitors may occupy the endosteal and perivascular niches of normal haematopoietic stem/progenitor cells (HSCs and HPCs) [45, 55, 72, 87, 128, 129]. More particularly, the homing of primitive LSCs in the hypoxic microenvironment in BM endosteum, which may lead to an up-regulation of HIF-1α and metabolic adaptation of these immature leukaemic cells to low oxygen supply and glucose availability as observed for their normal counterpart, long-term HSCs (LT-HSCs), may contribute to their survival and treatment resistance [6, 55, 56, 69, 90, 128–130]. In fact, the homing of LSCs under a quiescent state in the more hypoxic endosteal region of BM may allow these immature leukaemic cells to exist in a low cycling state that protects them from oxidative stress, DNA damages and cell death stimuli induced by cytotoxic agents [45, 87, 90, 94, 129].

More particularly, chronic myeloid leukaemia (CML) is a clonal disorder which is often accompanied by a chromosomal translocation resulting in a shortened chromosome 22, designated the Philadelphia (Ph^+^) chromosome in HSCs [68–70]. This molecular event generates a chimeric BCR-ABL fusion oncoprotein endowed with a constitutive tyrosine kinase activity in LSCs in the initial chronic phase of CML [68–70]. Typically, this initial chronic phase of CML progresses to an accelerated phase mediated through the occurrence of further oncogenic events in LSCs, that ultimately leads to the terminal phase of CML, designated as blast-crisis (Bc)-CML [68–70, 131]. Also, an increase in the β-catenin level may occur in the granulocyte-macrophage progenitors when patients reach a later stage of Bc-CML and result in their acquisition of an enhanced self-renewal capacity and leukaemic potential [68, 130]. In this matter, it has been shown that tyrosine kinase inhibitors (TKIs) such as imatinib mesylate (IM) or the second generation of drugs, including dasatinib, nilotinib and bafetinib (INNO-406), targeting the BCR-ABL fusion protein in proliferative leukaemic cells are highly effective for treating patients with chronic phase CMLs and Ph-positive acute lymphoid leukaemias (ALLs) [6, 56, 91–93, 130, 132]. Unfortunately, CML cells can exhibit BCR-ABL kinase-dependent and -independent mechanisms of resistance to these TKIs [6, 56, 91–93, 130]. Moreover, these TKIs did not appear to eradicate the total mass of leukaemic cells including primitive and quiescent BCR-ABL LSCs with a high self-renewal potential [6, 56, 91–93, 130]. Consequently, the persistence of LSCs in the hypoxic endosteal niche of BM after cessation of TKI therapy may result in disease relapse and require a long-life treatment with TKIs for the survival of CML patients [6, 56, 91– 93, 130]. Importantly, it has been observed that human BCR-ABL^+^ CML-LSCs engrafted in the BM of immunodeficient mice and survived under severe hypoxia (<1.3% oxygen) along the endosteum [90]. It has also been shown that BCR-ABL fusion oncoprotein can up-regulated the expression levels of HIF-1*α* and its target genes *via* the stimulation of PI3K/Akt/mTOR pathway in the murine BCR-ABL^+^ Ba/F3 leukaemic cell line and contribute to their survival [55]. Moreover, hypoxia and HIF-1α in turn can promote the selection of LSCs in CML that are refractory to IM and bortezomib [90, 94, 133]. More specifically, it has been reported that hypoxia-adapted BCR-ABL^+^ leukaemic cell lines obtained after long-term culture under 1% oxygen level exhibited stem cell-like properties, a great number of leukaemic cells in the side population (SP) and under a dormant state, high resistance to TKIs including IM, INNO-406 or dasatinib, enhanced expression level of β-catenin and glyoxalase-1 activity and transplantation efficacy [90]. Hence, these data suggest that BCR-ABL induced–enhanced expression of HIF-1*α* in CML cells, including CML-LSCs, may contribute to their quiescence and survival in the hypoxic endosteal niche of HSCs in BM after treatment initiation and disease relapse.

Of therapeutic interest, the deletion of HIF-1α in a mouse model of human CML has been observed to inhibit the cell cycle progression and induce the apoptosis *via* an induction of p16^INK4A^ and p19^ARF^ tumour suppressor proteins in LSCs [55]. Moreover, it has been shown that a natural product of *Micromonospora* strain designated as Rakicidin A, which acts as a hypoxia-selective cytotoxin, was effective at inducing the apoptotic death of TKI-resistant and hypoxia-adapted BCR-ABL^+^ CML cells endowed with stem cell-like properties maintained in suspension under low-oxygen conditions for more than 6 months [56]. The combined use of Rakicidin A plus IM or dasatinib also resulted in synergistic cytotoxic effects on hypoxia-adapted BCR-ABL^+^ CML cells [56]. In the same way, it has also been observed that the down-regulation of HIF-1α by small hairpin RNA (shRNA) or using a HIF-1α inhibitor, echinomycin eradicated mouse lymphoma-initiating cells and human acute myeloid leukaemia (AML)-LSCs in both *in vitro* colony formation assays and *in vivo* mouse models, whereas normal HSCs were 100-fold less sensitive to echinomycin than lymphoma CSCs *in vitro* [134].

On the other hand, other molecular mechanisms that may contribute to the resistance of LSCs or their early progenitors also include the elevated expression of β-catenin and enhanced glycolytic metabolism and autophagy under normoxic and hypoxic conditions [90, 130, 135]. Importantly, a novel Wnt/β-catenin signalling inhibitor, AV65, has been reported to reduce the β-catenin expression and inhibit the proliferation of IM-resistant and hypoxia-adapted CML cells [130]. It has also been noted that a combination of AV65 plus IM induced synergistic anti-proliferative effects on CML cells [130]. Moreover, the targeting of glyoxalase-1, which catalyses the detoxification of a highly cytotoxic by-product of glycolysis termed methylglyoxal, using a specific inhibitor termed *S*-*p*-bromobenzylglutathione cyclopentyl diester was also more effective at inducing the apoptotic effects on TKI-resistant, quiescent and hypoxia-adapted BCR-ABL leukaemic cells with acquired stem cell-like features than on parental leukaemic cell lines *in vitro* and *in vivo* [90]. In the same way, the inhibition of autophagy by using pharmacological agent such as bafilomycin A1 or 3-methyladenine as well as chloroquine which can act as an inhibitor of late-stage autophagy has been observed to potentiate the cytotoxic effects induced by TKIs such as IM or dasatinib on CML cells, including more primitive CML-LSCs, *in vitro* and *in vivo* [135].

Overall, these recent studies have underlined the critical role of hypoxia and H1F-1α, altered metabolic pathways and autophagy for the survival and treatment resistance of LSCs in the hypoxic microenvironment of BM, including in the insensitivity of BCR-ABL^+^ CML-LSCs to TKIs that target the bulk mass of proliferative leukaemic cells. Thus, the combined inhibition of H1F-1α, glycolysis and autophagy constitutes promising approaches to kill the LSC subpopulation in the hypoxic endosteal niche of BM that may be responsible for treatment resistance and disease relapse, and thereby improve the efficacy of current therapies against aggressive and recurrent leukaemias.

## Functions of hypoxia and HIFs in the development of glioblastoma multiforme and targeted therapies

Glioblastomas are among the most frequent, aggressive and lethal brain tumours because their rapid progression to locally invasive disease states and development of different molecular mechanisms of resistance by tumour cells to current radiation therapies and chemotherapies with DNA-alkylating agents such as temozolomide, nitrosoureas and/or cisplatin [82, 136–138]. In this regard, recent accumulating lines of evidence have revealed that a subpopulation of glioma stem cells (GSCs), also designated as glioma-initiating cells, expressing stem cell-like markers such as CD133, nestin, CD44, Oligo-2, Oct-3/4, Sox-2, Nanog, Musashi and/or Bmi-1 and endowed with high self-renewal and tumourigenic capacities may be responsible for driving glioblastoma multiforme (GBM) development, local invasion, resistance to current therapeutic treatment and disease recurrence [82, 126, 127, 139–141].

Glioblastoma multiforme is a heterogeneous disease and encompasses distinct molecular subtypes characterized by specific gene signatures [82, 142–144]. In general, GBMs are highly vascularized tumours and exhibit intratumoral heterogeneity, including in phenotypic features of tumour cells found within normoxic and hypoxic regions [1, 82, 127]. Particularly, GBM pathogenesis typically implicates an increased expression of many growth factors, cytokines and chemokines and their cognate receptors in tumour cells that cooperate to their malignant transformation and acquisition of more aggressive phenotypes and tumour neoangiogenesis during progression to locally invasive GBMs [82]. The changes within local tumour microenvironment of GBM cells, including hypoxia also may induce the enhanced expression of HIF-1α, HIF-2α and stem cell-like markers such as pluripotency-associated gene products Oct-3/4, Sox-2, Nanog and Myc that promote their stem cell-like properties and gain of a more aggressive behaviour [126, 127, 145, 146]. Importantly, immunohistochemical analyses have indicated that HIF-1α and HIF-2α and their target genes, including glycolytic enzymes and VEGF are frequently overexpressed in tumour cells in hypoxic zones closest to areas of necrosis, which demarcate surrounding regions of tumour angiogenesis, during GBM development [1, 2, 127]. Moreover, it has been noticed that an enhanced expression of HIF-1α and HIF-2α in tumour cells was strongly correlated with tumour grade and vascularity of GBM tissue specimens [1]. These data suggest that HIF-1α and HIF-2α can contribute to the rapid re-oxygenation of hypoxic zones of GBMs that may be mediated in part through the enhanced expression of pro-angiogenic factors such as VEGF (Fig. 2). In this regard, it has also been observed that GSCs are enriched in a region, designated as vascular niche around tumour vessels as well as in a hypoxic niche localized near necrotic areas associated with restricted oxygen and nutrients [82, 139–141]. It has also been shown that HIF-2α can play key roles in GSCs found within perinecrotic/hypoxic niche by inducing enhanced expression of specific tumour stem cell signature genes, including mastermind-like protein 3 (Notch pathway), nuclear factor of activated T cells 2 (calcineurin pathway) and aspartate β-hydroxylase domain-containing protein 2 that are associated with a poor prognosis of GBM patients [147]. In the same way, the enhanced expression of HIF-1α in CD133^+^ GSCs from human glioma specimens propagated under hypoxia (1% oxygen) also promoted their self-renewal capacity, inhibited their differentiation and led to the expansion of GSCs expressing CXCR4, CD44^low^ and A2B5 surface markers [148].

Interestingly, it have also been observed that GSCs expressing stem cell-like markers CD133, Oligo-2, Oct-3/4, Sox-2, Nanog, Musashi and Bmi-1 from human glioblastoma biopsy specimens xenografted into brains of immunocompromised mice secreted high levels of VEGF, which were promoted under hypoxia, and generated highly vascularized tumours with areas of necrosis and haemorrhage [149]. In contrast, CD133^−^ tumour cells only formed a few secondary tumours that were poorly vascularized [149]. In the same way, the data of immunohistochemical analyses have indicated that HIF-2α was co-expressed with CD133 stem cell-like marker in GBM tissue specimens, and associated with a poor survival of glioma patients [127]. It has also been noted that HIF-2α and its target genes, including Oct-3/4, SerpinB9, GLUT-1 and VEGF were preferentially expressed in CD133^+^ GSCs from glioblastoma surgical biopsy specimens or xenografts derived from brain tumour specimens as compared with CD133^−^ tumour cells under hypoxic conditions [127].

In addition, the maintenance of GSCs and their differentiated progenies in normoxic and hypoxic regions within tumours also requires the interplay of different intrinsic and extrinsic factors that govern the embryonic brain tumour development [41, 82, 127, 150, 151]. Particularly, the sustained activation of different growth factor cascades, including EGFR, constitutively activated EGFRvIII mutant and IGF-1/IGF-1R may stimulate PI3K/Akt/mTOR signalling elements that in turn induce the stabilization and/or translation of HIF-1α and HIF-2α proteins in GSCs and their progenies and angiogenic switch [82, 137, 152]. Moreover, GBM development is typically characterized by a high incidence of *PTEN* mutation in late stage of tumorigenesis [41, 82, 137, 153]. *PTEN* inactivation may promote the sustained activation of PI3K/Akt pathway that in turn can induce HIF-1α stabilization and transcriptional expression of its target genes, and thereby contribute to the survival of GBM cells under normoxic and hypoxic conditions [41, 82, 137, 153]. For instance, it has been observed that the ectopic overexpression of wild-type PTEN in the human U373 glioblastoma-derived cell line lacking functional PTEN reduced IGF-1- and hypoxia-induced Akt activation and consequently the HIF-1α stabilization and expression of its target gene products, including glycolytic enzymes, PGK-1 and PFK, COX-1 and VEGF [41].

Of therapeutic interest, numerous studies have also indicated the benefit to target HIF signalling network, including growth factors implicated in the regulation of HIF activity, altered metabolic pathways and angiogenic factors to kill GSCs and their differentiated progenies under normoxic and hypoxic conditions, and thereby prevent GBM progression to locally invasive disease state [108, 127, 148, 154, 155]. For instance, it has been observed that the targeting of HIF-1α or HIF-2α by shRNA in CD133^+^ GSCs from a patient's glioblastoma specimen inhibited their neurosphere-forming ability and proliferation, induced the caspase-dependent apoptotic effect *in vitro* and attenuated their tumour-initiating potential *in vivo* [127, 148]. Moreover, the down-regulation of histone methyltransferase mixed-lineage leukaemia 1 (MLL1) by shRNA in GBM cells, which is induced under hypoxic condition, was also effective at reducing the expression of HIF-2α protein and its target genes, including VEGF and inhibiting the self-renewal, growth and tumourigenicity of GSCs [108]. The targeting of VEGF using neutralizing mAb, bevacizumab was also effective at inhibiting tumour growth of xenografts derived from CD133^+^ glioma-initiating cells or U87 glioma cells by decreasing the number of self-renewing and vessel-associated CD133^+^/nestin^+^ tumour cells [141, 149].

In addition, the down-regulation of key regulators of autophagy such as DNA-damage regulated autophagy modulator protein 1 (DRAM1) or p62 encoded by sequestosome 1 (*SQSTM1*) by shRNA in GSCs has also been shown to impair autophagy, reduce metabolic energy, including ATP and lactate levels, and inhibit the migration and invasion of GSCs [154]. Moreover, the data from a randomized and double-blind, placebo-controlled trial carried out with 30 patients with surgically confirmed GBMs have also indicated that the autophagy inhibitor, chloroquine improved the anticarcinogenic efficacy induced by conventional radiotherapy and chemotherapy and median survival of GBM patients [156].

Collectively, these observations suggest that HIF-induced–enhanced glycolysis and VEGF expression as well as altered autophagy in GSCs may represent selective adaptations that are necessary for their survival under stressful conditions, including in a hypoxic microenvironment and nutrient deprivation, rapid tumour neovascularization and re-oxygenation and progression to locally invasive disease states. The development of new combination therapies consisting to target HIF signalling network and altered metabolic pathways in GSCs and their progenies as well as the components of their vascular and hypoxic niches represents new therapeutic strategies to improve the efficacy of current therapies and counteract the progression to highly invasive and lethal GBMs.

## Functions of hypoxia and HIFs in the development of melanoma and targeted therapies

Malignant cutaneous melanoma is the most aggressive and lethal form of skin cancer with a poor prognosis for patients with advanced disease [157–160]. Although surgical tumour excision of early melanocytic lesions is associated with high cure rates, the rapid progression to locally invasive and metastatic melanomas that are resistant to current radiotherapies and chemotherapies usually led to disease relapse and the death of melanoma patients [157–160]. A growing body of evidence has indicated that intertumoral and intratumoral heterogeneity of melanomas may be due in part to the differences between melanoma stem/progenitor cells at the origin of a particular melanoma subtype as well as the changes in their number and phenotypic and functional features during melanoma progression and after treatment initiation. In support with this, a subset of melanoma stem/progenitor cells expressing stem cell-like markers such as CD133, nestin, neural crest nerve growth factor receptor CD271, Oct-3/4, Nanog, multi-drug resistance protein-1 (MDR1), ABCG2 and/or ABCB5 has been isolated from human primary and metastatic melanoma specimens or melanoma cell lines [83, 161–165]. Tumourigenic melanoma stem/progenitor cells with high self-renewal capacity and aberrant differentiation potential were able to generate melanomas when transplanted into human skin or bone or animal models with histopathological features resembling human melanomas [161–164].

Importantly, high expression levels of HIF-1α, HIF-2α and their target genes such as VEGF, have also been detected in melanoma cells in up to 80% cases of primary and metastatic melanoma specimens from patients, and more particularly at the margin of necrotic areas of tumours, and associated with melanoma progression as well as a poor prognosis of patients [1, 166–169]. Moreover, a nuclear staining for pluripotency-associated transcription factor Oct-3/4 was also seen in a small subset of melanoma cells, and particularly in hypoxic cancer cells near necrotic areas within primary melanomas in vertical growth phase or metastatic melanoma tissue specimens [112]. It has also been shown that the exposure of melanoma cell lines to hypoxia up-regulated expression levels of HIF-1α and HIF-2α and their target genes including Oct-3/4, Nanog, Nodal, connective tissue growth factor (CTGF), snail-1 and VEGF that in turn promoted their self-renewal ability, tumourigenicity, metastatic potential and resistance to current chemotherapeutic drugs such as temozolomide and cisplatin as well as angiogenic switch [112, 170–172]. The ectopic overexpression of Oct-3/4 in melanoma cell lines was also effective to enhance their expression of stem cell-like markers such as CD271, MDR1, ABCG2, ABCB5, Kruppel-like factor 4 (KLF4) and Nanog and self-renewal capacity [112].

In addition to hypoxia, enhanced expression and/or stability of HIFs in melanoma cells may also be induced by stress signals such as heat shock and reactive oxygen species (ROS), microphthalmia-associated transcription factor (MITF) and stimulation of oncogenic growth factor cascades under normoxic and hypoxic conditions [173–180]. The tumourigenic signalling elements involved in the modulation of HIF activity include Notch, endothelins (ETs)/ET_B_ receptor and constitutively activated B-Raf or N-Raf mutant that may contribute to the sustained activation of PI3K/Akt, MAPKs and/or NF-κB [173–180]. Particularly, it has been shown that B-Raf^V600E^ mutant may induce an increase in HIF-1α expression under normoxic and hypoxic conditions [177]. It has also been noticed that the expression of a HIF-1α^785^ isoform lacking a part of the oxygen regulation domain and which is more stable than full-length HIF-1α under high oxygen tension, was also induced by 12V-H-Ras, hyperthermia, serum, EGF, phorbol 12-myristate 13-acetate (PMA), heat and oxidative stresses in melanoma cell lines under normoxic conditions [178, 181]. Moreover, tumour hypoxia has also been associated with enhanced expression of HIFs, lactate dehydrogenase 5 and autophagy-related proteins, including beclin-1 and light chain 3A (LC3A), in hypoxic melanoma cells that may promote anaerobic glycolysis and extensive autophagy activity and contribute to their survival under oxygen and nutrient deprivation [169]. On the other hand, high level of HIF-1α has also been detected in melanoma cells expressing melanoma antigen recognized by T cells-1 MART-1^+^ found in perivascular regions within tumour as well as MART-1^−^ non-haematopoietic melanoma-associated stromal cells, including CD146^+^ pericytes associated with CD31^+^ endothelial cells and Sca-1^+^ stromal cells in a mouse model of melanoma [182]. This suggests that stromal cells and melanoma cells, which can form functional vessel-like structures by vasculogenic mimicry, can cooperate for tumour vascularization supporting the interest to co-target these cells to counteract melanoma development.

Novel therapeutic strategies against aggressive and metastatic melanomas have also been investigated that consist to the molecular targeting of HIFs and/or their target gene products including pro-angiogenic factors such as VEGF, EMT programme- and altered metabolism-associated molecules in melanoma stem/progenitor cells and their progenies to counteract melanoma progression and metastases and reversing treatment resistance [23, 33, 172, 176, 179, 180, 183–188]. For instance, the ET_B_R blockage using a pharmacological antagonist, A-192621 resulted in a decrease in HIF-1α and HIF-2α stability concomitant with an increase in prolyl hydroxylase domain protein 2 level that was accompanied by an inhibition of tumour growth and angiogenesis of human M10 melanoma cell-derived xenografts in nude mice [176]. It has also been shown that the treatment of human A2058 melanoma cells with a small-molecule STAT3 inhibitor, CPA-7 was effective at inhibiting the expression of HIF-1α and VEGF *in vitro* and tumour growth and angiogenesis of human A2058 melanoma tumours *in vivo* [33].

Together these studies have indicated that the enhanced expression of HIFs and their target gene products and altered metabolism in melanoma cells during disease progression may promote their malignant reprogramming, including their acquisition of stem cell-like properties and more aggressive and metastatic phenotypes and angiogenic switch. The targeting of HIF signalling elements that are involved in the survival of melanoma-initiating cells under hypoxic and stressful conditions represents new promising strategies to prevent melanoma development and treatment resistance.

## Functions of hypoxia and HIFs in the development of prostate cancer and metastases

Prostate cancer (PC) is among the most common malignancies in men [160, 189, 190]. Metastatic PCs still represent the second leading cause of cancer-related death [160, 189, 190]. Although important advances have led to an earlier diagnosis and effective therapeutic intervention by prostatectomy and/or radiation therapy for patients with localized PCs, the disease progression to locally invasive and metastatic castration-resistant prostate cancers (CRPCs) is generally associated with treatment resistance and disease relapse [160, 189, 190]. Moreover, current anti-hormonal treatments and first-line docetaxel-based chemotherapies against metastatic CRPCs are only palliative and culminate in the death of most patients after about 12–19 months [160, 189, 190]. Importantly, accumulating lines of experimental evidence have indicated that PC- and metastasis-initiating cells expressing stem cell-like markers such as CD133^+^, CD44^high^, ALDH^high^, ABCG2^+^ and/or CXCR4^high^ and endowed with a high self-renewal ability can play critical functions for PC progression, metastases and resistance to current clinical therapies [45, 53, 95, 97, 191–194].

Recent studies have also revealed that an increase in expression levels and transcriptional activity of HIF-1α and HIF-2α frequently occur in PC cells during primary PC progression and bone metastases and is associated with treatment resistance and a poor outcome of patients [1, 4, 9, 13, 101, 195–200]. More specifically, the sustained activation of EGF and TGF-α/EGFR and TGF-β/TGF-βR cascades as well as the down-regulation or loss of PTEN and enhanced levels of inflammatory cytokines such as TNF-α during PC progression and after treatment initiation may result in the stimulation of PI3K/Akt/mTOR, NF-κB and/or MAPK signalling elements in PC cells [28, 34, 35, 42, 119, 201]. These tumourigenic pathways in turn may induce the expression, stabilization and nuclear translocation of HIF-1α and/or HIF-2α in PC cells under normoxic and hypoxic conditions and contribute to their gain of a more malignant behaviour [28, 34, 35, 42, 119, 201–203]. Moreover, it has been observed that the changes in the tumour microenvironment of PC cells, including the prevalence of hypoxic zones in primary PCs, and more particularly at the invasive front, may induce HIF-1α and HIF-2α and the reprogramming of PC cells to re-express high levels of some stemness gene products such as CD44, Oct-3/4 and Nanog (Figs 2 and 3) [4]. The maintenance of PC cell lines under hypoxic conditions has also been observed to result in their acquisition of more aggressive phenotypes and enrichment of hypoxic cancer cells with stem cell-like features and expressing high levels of drug resistance-associated molecules, such as multi-drug transporter, ABCG2 and anti-apoptotic factor, Bcl-xL [8, 14, 48, 57, 111, 204]. For instance, it has been observed that the culture of human androgen-independent (AI) and metastatic PC3 and DU145 prostatic cancer cells under low oxygen level (7% or 1% O_2_) was accompanied by up-regulation of HIF-1α and HIF-2α expression and enhanced colony formation efficacy of these hypoxic PC cells as compared with normoxic conditions (20% O_2_) [14]. Moreover, the number of SP cells and PC3 and DU145 cells expressing stem cell-like markers such as CD44^high^, ABCG2^high^, Oct-3/4 and Nanog and endowed with high prostasphere-forming ability detected in PC3 and DU145 cell lines was also increased under hypoxia [14]. On the other hand, the conditioned medium from PC-associated fibroblasts activated by TGF-β1 or IL-6 has also been observed to promote in a paracrine manner the induction of EMT programme in PC3 cells and their gain of stem cell-like features, including high prostasphere-forming capacity and migratory ability by inducing inflammatory factors such as cycloxygenase-2 (COX-2), NF-κB and HIF-1α [205, 206].

In addition, some studies have also indicated that the metastases of PC cells at distant sites, including bones, may occur early during disease progression, and the persistence of dormant PC cells homing at bones, may represent a determinant factor associated with the bone tumour formation and disease relapse after treatment initiation (Fig. 4) [53, 207–209]. Some similarities between the molecular mechanisms that govern the specific engraftment and homing of HSCs to bones are also observed for PC cells that preferentially migrate and adhere to bones. More specifically, the chemoattractant gradient formed by SDF-1 molecules released from BM-resident endothelial cells and stromal cells appears to provide critical functions for the specific dissemination, engraftment, migration through matrix and homing of CXCR4^+^ PC cells to BM as observed for LT-HSCs (Fig. 4) [53, 208, 210–214]. Moreover, it has been shown that metastatic PC cells homing at BM can compete with resident HSCs to occupy the most hypoxic endosteal niche formed by osteoblasts [208, 211]. In this regard, numerous growth factors and cytokines, including SDF-1, EGF, sonic hedgehog (SHH), TGF-β1, bone morphogenic proteins (BMPs) and MIC-1 released by metastatic PC cells, stromal fibroblasts, osteoblasts, osteoclasts and HSC/HPCs in BM microenvironment may contribute to the regulation of dormancy phenomenon of PC cells in hypoxic endosteal niche and promote their re-activation and formation of macrometastases and osteoblastic and/or osteolytic lesions under specific microenvironmental conditions [53, 194, 209, 215–217]. More particularly, it has been shown that BMP-7 secreted by BM-resident stromal cells may activate BMP receptor 2-induced p38/N-myc downstream-regulated gene 1 axis and cause a reversible senescence state in PC stem cell-like cells suggesting that BMP-7 can play a critical role in the regulation of their dormancy and hibernation at bones [216]. Moreover, it has also been observed that CXCR4 was highly up-regulated in both metastatic and AI PC3 and DU145 cells grown under prostasphere-forming conditions compared with monolayer growth conditions as well as in the CD133^+^/CD44^+^ PC stem/progenitor cell subpopulation from these PC cell lines relative to the CD133^−^/CD44^−^ PC fraction [194]. The isolated CXCR4^+^ or CD133^+^/CD44^+^/CXCR4^+^ PC3 and DU145 stem/progenitor cell subpopulation also displayed higher prostasphere- and colony-forming abilities *in vitro* and tumourigenicity *in vivo* than CXCR4^−^ or CD133^−^/CD44^−^ PC cells [194]. It has also been observed that the stimulation of PC3 and DU145 cells with exogenous SDF-1 activated PI3K/Akt-induced inhibition of forkhead (FOXO3A) transcription factor pathway and led to an enrichment of CD133^+^/CD44^+^ PC cell number (Fig. 3) [194]. These data suggest that the stimulation of CD133^+^/CD44^+^/CXCR4^+^ PC stem/progenitor cells by SDF-1 can induce the PI3K/Akt cascade that in turn plays critical functions for their high self-renewal and skeletal metastases. Future investigations are, however, necessary to further establish the functions of the hypoxic microenvironment in the BM endosteal niche and HIFs in controlling the dormancy phenomenon, self-renewal, survival and formation of well-established metastases by metastasis-initiating PC cells as well as their interactive cross-talks with other growth factor pathways including SDF-1/CXCR4 axis and TGF-β family members.

### Novel therapies by targeting HIFs and altered metabolic pathways in PC stem/progenitor cells and their differentiated progenies

Numerous studies have been carried out to establish the therapeutic benefit to down-regulate expression levels, stability and/or transcriptional activity of HIF-1α and/or HIF-2α by RNA interference or using pharmacological inhibitors of HIFs to eradicate PC cells [8, 54, 57–61, 202, 206, 218–222]. Among the pharmacological agents targeting HIF signalling network, there are specific inhibitors of HIFs (YC-1, P276-00, P3155, JG243, JG244 and NSC-134754), zinc, cyclin-dependent kinase inhibitor (P276-00), histone deacetylases (panobinostat) and mTOR complex 1 (everolimus) (Table 1) [8, 54, 57–61, 206, 218–222]. The results have indicated that the targeting of HIF pathway with these inhibitory agents induced the anti-proliferative, anti-invasive, anti-metastatic and/or apoptotic effects on PC cells under normoxic and hypoxic conditions and improved the cytotoxic and anti-angiogenic effects induced by irradiation and chemotherapy *in vitro* and *in vivo* [8, 54, 57–61, 202, 206, 218, 219, 221, 222]. For instance, it has been reported that the prostasphere-forming capacity of PC3 cells stimulated by the conditioned medium from activated fibroblasts as well as their tumour growth and metastatic spread in nude mice were significantly inhibited by shRNA targeting the pro-inflammatory signature including COX-2, NF-κB or HIF-1α [206]. New G-rich ODNs termed JG243 and JG244, which form an intramolecular parallel G-quartet structure, have also been observed to selectively interact with HIF-1α and HIF-2α proteins and induce their proteasomal degradation [54]. JG243 or JG244 mixed with a solution of polyethylenimine (PEI) was also effective at inhibiting the expression of HIF-regulated proteins such as VEGF, Bcl-2 and Bcl-xL and dramatically suppressing the growth of human PC3 tumour xenografts in nude mice [54]. Moreover, a combination of JG244/PEI plus a G-rich ODN directed against the phosphorylated STAT-3 protein termed T40214/PEI also inhibited the growth and induced the apoptotic effect on human DU145 prostate tumours and transgenic adenocarcinoma of mouse prostate (TRAMP)-C2 model *in vivo* as compared with drugs alone [218]. Interestingly, it has also been observed that the treatment of PC3 cells with anthracyclines, doxorubicin or daunorubicin was effective at suppressing the HIF-1α transcriptional activity and its target genes, GLUT-1 and VEGF [58]. Also, treatment of severe combined immunodeficient (SCID) mice bearing PC3 cell-derived tumour xenografts with doxorubicin or daunorubicin significantly reduced the tumour growth, recruitment of BM-derived cells and angiogenesis as compared with untreated mice [58]. Another study using doxorubicin encapsulated in pegylated liposomes has also indicated that this liposomal formulation Caelyx® was effective at improving the cytotoxic effects induced by radiation treatment on hypoxic tumours derived from androgen-sensitive CWR22 cells [201]. These data suggest that anthracyclines could be used, either alone or in combination therapy with current chemotherapeutic drug docetaxel, to inhibit the HIF-1α activity and induce the anti-angiogenic effects in hypoxic prostate tumours.

In considering the fact that the major cause of disease relapse of PC patients is caused by a rapid spread of PC cells to distant sites, including bones, and their homing under a dormant state, the targeting of metastasis-initiating PC cells is highly necessary to improve the efficacy of current cancer treatments and prevent severe and intractable pain associated with osteoblastic and osteolytic bone lesions (Fig. 4). Importantly, it has been observed that the enhanced expression of CXCR4 in PC cells, which may be induced under hypoxic conditions, was associated with a high risk of metastases at distant sites including bones and poor outcome of cancer patients [223]. Of therapeutic interest, it has also been reported that the targeting of CXCR4 using antagonist AMD3100 or anti-CXCR4 antibody was effective at decreasing the CD133^+^/CD44^+^ PC subpopulation, but did not significantly affect the CD133^−^/CD44^−^ PC fraction within the total mass of PC3 or DU145 cells [194]. An opposed effect, however, was seen with docetaxel or 5-fluorouracil treatment which induced an enrichment of CD133^+^/CD44^+^ PC subpopulation [194]. Importantly, a combination of CXCR4 antagonist AMD3100 or Akt inhibitor NVP-BEZ235, which targets CD133^+^/CD44^+^/CXCR4^+^ DU145 stem/progenitor cells plus docetaxel was also more effective at inducing the tumour growth inhibitory effect on DU145 cell xenografts in non-obese diabetic (NOD)/SCID mice and preventing the tumour re-growth after treatment cessation as compared with individual drugs [194]. Moreover, the inhibition of SDF-1/CXCR-4 axis in metastatic PC3 cells using anti-CXCR4 monoclonal antibody (mAb) or CXCR4 antagonist, AMD3011 has been observed to impair their homing at the hypoxic endosteal niche in BM and inhibit bone tumour formation [208, 211, 224]. These results underline great interest to target CXCR4 and Akt in PC- and metastasis-initiating cells for improving current therapies and preventing disease relapse.

On the other hand, numerous studies have also revealed the possibility to inhibit lipogenesis, glycolysis and/or autophagy to restraint the energy supply required for a high proliferation rate and survival of metastatic and hypoxic PC cells including PC stem/progenitor cells [48, 95, 225–234]. Particularly, the data from global transcriptional profiling have revealed that the activity of monoacylglycerol lipase (MAGL), which plays a major role in lipogenesis in metastatic PC cells by converting monoglycerides to free fatty acids, was higher in AI and metastatic PC3 and DU145 cells relative to androgen-dependent LNCaP cells and associated with a gene signature that correlated with the EMT programme and stem cell-like properties of PC cells [234]. Also, the treatment of PC3 and DU145 cells with a selective inhibitor JZL184 of MAGL activity was effective at reducing their migration, invasion and survival *in vitro* and inhibiting the tumour growth of PC3 cell xenografts in SCID mice [234]. In addition, the results from a phase I trial have also indicated that the administration of a synthetic glucose analogue, 2-deoxy-D-glucose (2-DG) to PC patients had no major secondary effects and five of eight patients assessed with fluorodeoxyglucose (^18^F)-positron emission tomography (FDG-PET) scanning exhibited a decreased FDG uptake by day two of therapy [228]. It has however been noted that a treatment with 2-DG may be associated with enhanced autophagy in PC cells which may contribute to their 2-DG resistance [228]. Of therapeutic interest, the combination of 2-DG with an anti-diabetic compound including pioglitazone or metformin, which acts at least in part as an inhibitor of 2-DG-induced autophagy, has been observed to be more effective at inducing the apoptotic effects on metastatic LNCaP, PC3 and DU145 cells than drugs alone [229, 232].

Collectively, these recent studies have revealed that the adaptation of AI and metastatic PC stem/progenitor cells and their differentiated progenies to hypoxia and nutrient deprivation through the induction of HIFs, glycolytic pathways and autophagy may result in their enhanced expression of pluripotency-associated molecules and acquisition of a more aggressive behaviour during PC progression and bone metastases. Novel inhibitors of HIF-1α and/or HIF-2α and altered energy metabolism have been shown to be effective at inducing cytotoxic effects on hypoxic PC cells. Thus, these observations support therapeutic interest to further investigate these pharmacological agents for eradicating hypoxic PC- and metastasis-initiating cells endowed with stem cell-like properties and reversing the resistance to current anti-hormonal treatments, radiation therapy and docetaxel-based chemotherapies.

## Functions of hypoxia and HIFs in the development of breast cancer and metastases

Breast cancer encompasses a heterogenous group of disease characterized by an accumulation of different genetic and epigenetic alterations occurring in the basal and/or luminal breast epithelial cells in the mammary gland [235, 236]. Although breast tumour resection may lead to a high survival rate for breast cancer patients diagnosed at early stages, locally advanced and highly invasive and metastatic breast cancer subtypes are generally refractory to current anti-hormonal treatments, targeted therapies against erbB2/HER2, irradiation and chemotherapies [160, 237, 238]. The heterogeneous nature of breast cancers may be due in part to the implication of different subpopulations of breast cancer stem cells (BCSCs) and their early progenitors that are responsible for tumour development and metastases at distant sites [73–80, 88, 239]. More specifically, highly tumourigenic BCSCs and their early progenitors endowed with a high self-renewal potential and which can express different stem cell-like markers such as CD44^+^, CD24^−/low^, epithelial-specific antigen (ESA^+^), CD133^+^, ALDH1^high^, Oct-3/4, Nanog, Kruppel-like factor (KLF-4) and/or CXCR4^high^ have been detected and isolated from breast tumour specimens from patients and breast cancer cell lines [73–80, 88, 239]. Moreover, gene expression profiling and gene set enrichment analyses have revealed that CD44^+^/CD24^−/low^ BCSC subpopulation showed increased expression of genes involved in TGF-β, TNF-α, interferon and NF-κB pathways that can contribute to the induction of the EMT programme in BCSCs and promote their mammosphere-forming ability and tumourigenicity [239–245]. Importantly, BCSCs expressing high levels of multi-drug transporters such as brain cancer resistance protein (BCRP)/ABCG2^high^, DNA repair enzymes and free radical scavengers and which can survive under oxygen and nutrient deprivation have been shown to be involved in the resistance to anti-hormonal and anti-angiogenic treatments, radiation and chemotherapies [45, 74, 79, 96, 241, 246–250].

Recent lines of experimental evidence have also indicated that enhanced expression levels of HIFs in BCSCs and their differentiated progenies in hypoxic intratumoral regions within poorly vascularized tumours and hypoxic BM microenvironment as well as the induction of the EMT programme may result in their acquisition of stem cell-like features, a high rate of glycolytic metabolism and more aggressive and invasive phenotypes as well as enhanced tumour angiogenesis during breast cancer progression [4, 15, 16, 50, 74, 75, 77, 79, 88, 251]. In support of this, a direct relationship between the co-expression of HIF-1α and the CD44^+^/CD24^−/low^ phenotype has been observed by immunohistochemical analyses of 253 samples of breast ductal carcinoma from patients, and associated with a worse prognosis of breast cancer patients [50]. Moreover, it has been observed that the expression levels of Jagged2 and nuclear Notch intracellular domain were up-regulated in hypoxic regions at the invasive front of breast tumour tissues, and the enhanced expression of Jagged2 in breast cancer cells cultured under hypoxia led to the activation of Notch pathway and induction of the EMT programme [15]. An enhanced activity of HIF-1α under hypoxia has also been observed to result in the up-regulated expression of TGF-β superfamily member, Nodal through the activated Notch pathway in breast cancer cells that in turn contributed to their invasion and metastatic spread [16]. Importantly, CD44^+^/CD24^−/low^ BCSCs expressing high levels of HIF-1α and mesenchymal markers such as N-cadherin and vimentin, but low level of E-cadherin, activated Wnt/β-catenin, and PI3K/Akt cascades also displayed higher clonogenic and mammosphere-forming abilities and tumourigenicity under normoxic and hypoxic conditions than their differentiated progenies [16, 74, 88]. Interestingly, an exposure of non-adherent human metastatic MDA-MB-231 and BCM2 breast cancer cells to three cycles of hypoxia and re-oxygenation has also been observed to be accompanied by an enrichment of the CD44^+^/CD24^−/low^/ESA^+^ BCSC fraction [79]. The CD44^+^/CD24^−/low^/ESA^+^ BCSC subpopulation from MDA-MB-231 and BCM2 cells also expressed the EMT markers such as vimentin and snail and decreased expression of E-cadherin and displayed higher mammosphere-forming capacity, tumourigenicity and metastatic potential to lungs as compared with parental breast cancer cell lines [79]. In addition, it has also been observed that activated breast cancer-associated stromal myofibroblasts may promote the mammosphere formation and tumourigenicity of breast cancer cells through the release of SDF-1 that in turn stimulates CD44^+^/CD24^−/low^ BCSCs expressing their cognate receptor CXCR4 and angiogenesis [252].

Although the molecular mechanisms that control the high propensity of breast cancer cells to preferentially metastasize to specific tissues and organs, such as lungs and bones remain not precisely established, it has been shown that hypoxic breast cancer cells within primary and secondary breast tumours can play critical roles in the formation of pre-metastatic niches and metastases within the hypoxic bone microenvironment (Fig. 4) [15, 239, 253–256]. In this matter, an increased expression level of HIF-1α in primary breast tumour and metastases has been associated with enhanced rates of metastases at distant sites and decreased survival of breast cancer patients [1, 10]. More specifically, it has been shown that HIF-1α may induce an enhanced expression of lysyl oxidase (LOX), lysyl oxidase-like 2 (LOXL2) and LOXL4 in hypoxic breast cancer cells within primary breast tumour [125, 257]. LOX and LOXLs secreted from hypoxic breast cancer cells in turn can contribute to the formation of pre-metastatic niches at distant tissues such as lungs by inducing the remodelling of the extracellular matrix (ECM) through cross-link collagens and elastins and promoting the recruitment of CD11b^+^ bone marrow-derived cells (BMDCs) [125, 257]. Moreover, the enhanced expression of CXCR4 in breast cancer cells can also play critical roles for their preferential metastatic spread to distant sites, including bones and lungs, which secrete high levels of SDF-1 ligand molecules that act as a chemoattractant gradient (Fig. 4) [256].

In addition, it has also been shown that BCSCs can be involved in bone metastases within hypoxic bone microenvironment [15, 253–255]. Particularly, different growth factors and cytokines released by stromal cells and breast cancer cells, including SDF-1, TGF-β1 and BMPs as well as the up-regulation of HIF-1α, NF-κB, vascular cell adhesion molecule-1 (VCAM-1) and Notch in breast cancer cells typically control their dormancy, survival and self-renewal ability and formation of osteolytic bone metastasis [1, 15, 239, 251, 254, 258–261]. More specifically, a novel animal model of breast cancer metastasizing to bone has been investigated which consisted of injecting human CD44^+^/CD24^−/low^ BCSCs subpopulation from MDA-MB-231 cells in mice previously implanted with human bone in the right or left dorsal flanks [253]. It has been observed that BCSCs displayed higher incidence of human bone metastasis relative to the parental breast cancer cell line, and metastatic bone tissues strongly stained for CD44, CXCR4 and osteopontin [253]. Moreover, it has also been noted that the enhanced activity of HIF-1α and TGF-β signalling elements promoted the EMT programme and up-regulated the expression levels of CXCR4 and VEGF in breast cancer cells, and thereby cooperated for their invasion, metastatic spread to bones and skeletal metastases [239]. In contrast, BMP-2/7 heterodimer, which acts as a powerful antagonist of TGF-β signalling pathway-induced EMT programme and invasiveness of breast cancer cells, strongly reduced the number of ALDH^high^/CD44^high^/CD24^−/low^ BCSCs and bone metastases [262]. Furthermore, the co-culture of CD44^+^/CD24^−/low^/ESA^+^ BCSCs from MDA231BoM cell line endowed with a strong propensity to metastasize to bones with immortalized human BM stromal cells HS5 expressing Jagged2 under hypoxia also resulted in the activation of Notch pathway in BCSCs that promoted their self-renewal potential [15]. These data suggest that the interactions between stromal cells and BCSCs in hypoxic BM microenvironment can play important functions for the regulation of their dormant state, self-renewal ability, bone metastases and treatment resistance. Hence, this underlines great interest to target hypoxic BCSCs and their supporting host cells in the hypoxic endosteal niche of BM to prevent skeletal metastases and disease relapse.

### Molecular targeting of HIFs and altered metabolic pathways in BCSCs and their differentiated progenies

In view of the fact that BCSCs appear to be principal cancer cells responsible for breast tumour development and metastases in the hypoxic bone microenvironment and are typically more resistant than their differentiated progenies to anti-hormonal and herceptin treatment, chemotherapy and radiotherapy, their molecular targeting is of major importance to prevent disease recurrence. In this regard, numerous studies have indicated that the targeting of HIF and altered metabolic pathways may eradicate hypoxic breast cancer- and bone metastasis-initiating cells, reduce tumour angiogenesis and improve the efficacy of current cancer therapies (Tables 1 and 2) [78, 96, 115, 239, 247, 249, 257, 263, 75, 264–267]. For instance, it has been observed that the down-regulation of HIF-1α by shRNA or pharmacological inhibition with 2-methoxyestradiol inhibited the angiogenesis, reduced the tumour development in bone derived from MDA-MB-231 breast cancer cells intracardially injected in nude mice and increased the mouse survival [239]. Moreover, the systemic administration of a combination of specific inhibitors of HIF-1α and TGF-βRI signalling elements, 2-methoxyestradiol plus SD-208, respectively, that target breast tumour cells and bone microenvironment, was also more effective at decreasing bone metastases of MDA-MB-231 breast cancer cells and osteoclastic bone resorption and stimulating the formation of bone mass than individual drugs [239]. On the other hand, the targeting of CAIX, which is induced by HIF-1α in breast tumour cells under hypoxia and involved in the pHi regulation also constitutes a promising therapeutic strategy. It has been observed that inhibition of CAIX by shRNA or using pharmacological agents such as sulphonamide compounds (CAI17, ureido-sulphonamide, U-104) or glycosyl coumarins (GC-204 and GC-205) induced the apoptosis and reduced the primary tumour growth and lung metastases of hypoxic breast cancer cells [115]. Moreover, the inhibition of LOX, which is induced by HIF-1α in hypoxic breast cancer cells at primary tumours by shRNA, was also effective at preventing the CD11b^+^ BMDC recruitment, pre-metastatic niche formation and metastatic growth of MDA-MB-231 breast cancer cells at lungs in a mouse model [257].

Other therapeutic strategies to eradicate BCSCs and their progenies may also include the targeting of signalling elements such as hexokinase-2, AMP-activated protein kinase (AMPK), Akt/mTORC1 and/or fatty acid synthase (FASN) that are involved in the regulation of glycolysis, lipogenesis and/or autophagy induced under hypoxia and nutrient deprivation (Fig. 3 and Tables 1 and 2) [78, 96, 247, 249, 263, 75, 264–269]. For instance, the treatment of orthotopic tumours established from hypoxic pre-conditioned MDA-MB-231 cells with EGFR-targeted nanoparticles (NPs) loaded with paclitaxel and lonidamine, which is an inhibitor of hexokinase-2 that catalyses the phosphorylation of glucose to yield glucose 6-phosphate during the glycolysis, has been observed to reduce the tumour growth relative to NPs loaded with single agent [75]. The anticarcinogenic effects of NPs were mediated in part through the down-regulation of the expression levels of HIF-1α, EGFR, P-glycoprotein (P-gp) and SCF [75]. Moreover, a potent and orally bioavailable AMPK activator designated as OSU-53 has also been observed to reduce the viability and clonogenic growth of triple-negative MDA-MB-231 and MDA-MB-468 breast cancer cells *in vitro* and *in vivo* through the inhibition of mTOR pathway, lipogenesis and HIF-1α-induced EMT programme [263]. It has however been noted that OSU-53 induced protective autophagy in breast cancer cells which could be attenuated by a co-treatment with an autophagy inhibitor, chloroquine that promoted the *in vivo* tumour-suppressive activity of OSU-53 [263]. Also, the inhibition of FASN activity, which is up-regulated in hypoxic regions of breast cancer tumours *via* the activation of Akt and HIF-1α-induced sterol regulatory element binding protein-1, using cerulenin or PI3K inhibitor LY294002, has also been observed to reverse the resistance of breast cancer cells to cyclophosphamide under hypoxic conditions [265]. In the same way, the CD44 knockdown using shRNA lentivirus particles in BCSCs also induced their differentiation and down-regulated the expression levels of FASN and different stem cell-like markers, oncogenes and anti-apoptotic factors such as lymphoid enhancer-binding factor-1 (LEF-1), Myc, EGFR, mucin-1 and Bcl-2 and thereby sensitized these immature cancer cells to the anti-tumoral effect induced by doxorubicin [249, 267]. Of particular interest, it has also been shown that anti-diabetic drug metformin inhibited different metabolic pathways, selectively eradicated BCSCs and acted in synergy with chemotherapeutic drugs such as doxorubicin and irradiation treatment to kill the total mass of breast cancer cells and thereby counteract tumour re-growth and disease recurrence [78, 96, 247, 250, 264, 269].

Altogether, these findings have indicated that the enhanced expression and activity of HIFs and altered metabolism in breast cancer cells can promote their malignant reprogramming and gain of stem cell-like features and thereby contribute to their high self-renewal ability, survival, tumourigenicity, invasiveness and treatment resistance under stressful conditions. Hence, novel multi-targeted therapies directed against HIFs and signalling elements involved in glycolysis, lipogenesis and autophagy, alone or in combination with current cancer therapies, constitute new promising approaches to eradicate total mass of breast cancer cells, including BCSCs, and thereby prevent their metastatic spread and disease relapse.

## Functions of hypoxia and HIFs in the development of pancreatic cancer and metastases

Pancreatic ductal adenocarcinomas (PDACs) are extremely aggressive solid tumours, with a poor 5-year survival rate of less than 6% [160, 270, 271]. The late stage detection and surgical resection of primary pancreatic tumours result in the disease relapse in most cases [160, 270, 271]. Moreover, the radiation therapy and first-line gemcitabine-based chemotherapeutic treatments of patients with locally advanced and metastatic PDACs are only palliative and lead to a modest improvement in survival rates [160, 270, 271]. The inefficacy of current treatments and the poor outcome of PDAC patients are because of a rapid progression to metastatic disease states and the development of diverse mechanisms of resistance by pancreatic cancer cells to conventional therapies [271–276]. Particularly, it has been shown that pancreatic cancer stem/progenitor cells expressing stem cell-like markers such as CD133, CD44 and ABCG2 multi-drug transporter may be more resistant to oxygen and nutrient deprivation, irradiation and gemcitabine treatment than the bulk mass of differentiated pancreatic cancer cells [12, 51, 52, 63, 275].

Pancreatic ductal adenocarcinomas are among the most hypoxic of all solid tumours, and are typically characterized by a dense stromal fibrosis also known as desmoplasia and a poor tumour vascularization that may contribute to restraint procedures of drug delivery and promote altered metabolic pathways and treatment resistance [11, 12, 274, 277–279]. In this regard, HIF-1α is up-regulated in PDACs and can play critical roles for the adaptation of pancreatic cancer cells and stromal cells to the hypoxic desmoplastic microenvironment during disease progression, treatment resistance and a poor outcome for PDAC patients [1, 11, 25, 118, 279–282]. Specifically, a nuclear HIF-1α staining was seen in about 88% of pancreatic ductal adenocarcinoma specimens from patients and 43% adjacent stroma, but in only 16% of the normal pancreatic tissues [11]. Moreover, the co-expression of HIF-1α and other important oncogenic products and drug resistance-associated molecules such as K-Ras mutant, which is detected in up to 75–90% of PDAC cases, hedgehog signalling elements, CXCR4, toll-like receptor 4 (TLR4), NF-κB p65, survivin, proliferating cell nuclear antigen and VEGF has also been detected in PDAC tissue specimens from patients and pancreatic cancer cell lines [118, 280, 282–288]. The expression levels of sonic hedgehog (SHH) signalling elements, which play critical functions in the desmoplasic lesion formation were also induced in pancreatic cancer cells under hypoxic conditions, and the tumour and stromal HIF-1α staining positively correlated with SHH ligand expression in pancreatic cancer tumour samples [285]. On the other hand, it has also been shown that the activation of IGF-1/IGF-1R and SCF/KIT axes in pancreatic cancer cells may contribute to the induction of HIF-1α through the stimulation of PI3K/Akt and/or Ras/MEK/ERK pathways and tumour angiogenesis under normoxic conditions [25, 26].

In addition, the data from immunohistochemical analyses have indicated that the markers associated with hypoxia (CAIX), pancreatic cancer stem/progenitor cells (CD44 and CD24) and autophagy (beclin-1 and microtubule-associated protein light chain 3 ‘LC3’) were co-expressed in PDAC tissue specimens from patients [51]. It has also been observed that the exposure of human MIA-PaCa-2 pancreatic cancer cells expressing high levels of CD44 and CD24 stem cell-like markers to hypoxia and nutrient starvation induced the EMT programme and the expression of HIF-1α and autophagy-related genes [51]. The hypoxia also enhanced the clonogenic capacity, survival, migration of MIA-PaCa-2 cells and formation of autophagic and acidic vesicles [51]. In contrast, BxPC-3 pancreatic cancer cells expressing low levels of stem cell-like markers did not survive under hypoxic and starvation conditions [51]. In the same way, the expression levels of CD133, CXCR4 and HIF-1α were also enhanced in the pancreatic cancer cell lines under hypoxia as compared with normoxic conditions and associated with an enhanced invasiveness of CD133^+^ pancreatic cancer cells [46, 51]. Importantly, the characterization of a series of early passage xenografts from 16 patients undergoing surgery for PDACs and orthotopically grown in nude mice has also revealed that the presence of hypoxic intratumoral regions was highly correlated with a rapid tumour growth and spontaneous metastasis formation [289]. Moreover, the analyses of the HIF-1α expression level in 48 pancreatic cancer tissues from patients who received adjuvant gemcitabine treatment after pancreatectomy have indicated that HIF-1α expression was associated with an enhanced neo-microvascularity in the hypoxic tumour environment and gemcitabine resistance [12]. It has also been noted that the patients with pancreatic tumours expressing a strong HIF-1α level had a shorter period until disease recurrence as compared with those with a weak HIF-1α expression underlining the importance of also targeting the HIF signalling network to kill hypoxic pancreatic cancer cells [12].

### Novel therapies by targeting HIFs and altered metabolic pathways in pancreatic stem/progenitor cells and their differentiated progenies

New therapeutic strategies by targeting hypoxia and HIF-1α pathways using RNA interference or specific inhibitory agents in pancreatic cancer- and metastasis-initiating cells and their differentiated progenies for improving current therapies have recently been investigated under normoxic and hypoxic conditions (Tables 1 and 2) [11, 62–65, 290]. For instance, it has been observed that a hypoxia-activated pro-drug designated as TH-302, which selectively targets hypoxic regions of solid tumours in combination with conventional chemotherapeutic drugs such as gemcitabine-induced greater anti-tumoral effects on diverse human tumour xenograft models including pancreatic cancer xenografts than individual drugs without major toxicity [64, 291]. Moreover, it has been reported that a novel fusicoccin derivative (ISIR-042) was more effective at inducing the growth inhibitory and cytotoxic effects on hypoxic pancreatic cancer cells than on normoxic pancreatic cancer cells *in vitro* and *in vivo* through a reduction in HIF-1α and Akt activation [63]. Also, ISIR-042 preferentially induced the cytotoxic effects on gemcitabine-resistant CD24^+^/CD44^+^ pancreatic cancer stem/progenitor cells from pancreatic cancer cell lines [63]. In the same way, the inhibition of HIF-1α by a novel selective inhibitor PX-478 was also effective at potentiating the cytotoxic effects induced by fractioned radiation treatment, with or without combined treatment with gemcitabine, on *in vitro* and *in vivo* human PANC-1, CFPAC-1 or SU.86.86 pancreatic cancer models at least in part by reversing radiation resistance of these hypoxic tumour cells and inhibiting the pro-angiogenic effect of HIF-1α [11].

Other potential strategies for eradicating pancreatic cancer stem/progenitor cells and their progenies and reversing treatment resistance, may consist of targeting Ras mutant, EGFR, IGF-1R, PI3K/pAkt and EMT process-associated molecules, altered metabolic pathways and autophagy under normoxic or hypoxic conditions [29, 30, 51, 63, 284, 292–296]. For instance, it has been reported that the pharmacological inhibition of NF-κB activity, which is activated in response to the enhanced expression and activity of HIF-1α under hypoxia, was effective at attenuating the induction of the EMT programme and reversing highly invasive and drug-resistant phenotypes of pancreatic cancer cells [296]. It has also been noted that the sensitivity of PANC-1 cells to gemcitabine was reduced under hypoxic conditions and the targeting of PI3K/Akt pathway using LY294002 plus human checkpoint kinase 1 (Chk1) inhibitor designated as 7-hydroxystaurosporine (UCN-01) partially reversed the gemcitabine resistance [274]. Of particular interest, the functional inhibition of active Ras by *S*-*trans*, *trans*-farnesylthiosalicylic acid was also effective at reducing HIF-1α expression and promoting anti-proliferative and apoptotic effects induced by the glycolytic inhibitor 2-DG on pancreatic cancer cells both *in vitro* and *in vivo* [284]. Importantly, the inhibition of autophagy using 3-methyladenine or monensin also reduced the clonogenicity, spheroid formation, expression of stem cell-like markers and tumourigenicity of pancreatic cancer cells and induced the apoptotic effect on pancreatic cancer cells with stem cell-like properties under hypoxic and starvation conditions [51]. Moreover, the anti-diabetic metformin, alone or in combination with difluorinated curcumin analogue (CDF), was also effective at inhibiting the cell survival, clonogenicity and pancreatosphere-forming ability of pancreatic cancer cells [276]. Metformin, alone or combined with CDF, also promoted the pancreatosphere disintegration in both gemcitabine-sensitive and gemcitabine-resistant pancreatic cancer cells [276].

Altogether, these observations suggest that the up-regulation of HIF-1α activity, glycolytic metabolism and autophagy may represent important adaptive processes for the survival of pancreatic cancer stem/progenitor cells and their progenies under tumour microenvironmental conditions such as hypoxia and nutrient deficiency. Consequently, the co-targeting of HIF-1α and altered metabolic pathways in pancreatic cancer- and metastasis-initiating cells and their differentiated progenies represent potential therapeutic strategies to counteract rapid PDAC progression, metastatic spread at distant sites, treatment resistance and disease relapse of this very aggressive and lethal disease.

## Conclusions and perspectives

Taken together, these recent investigations have revealed that the malignant reprogramming of cancer- and metastasis-initiating cells and their differentiated progenies may occur within hypoxic intratumoral regions at primary neoplasms and hypoxic niches at distant metastatic sites, including BM, and play critical roles for their acquisition of aggressive phenotypes and treatment resistance (Fig. 2). HIF-1α and HIF-2α appear to act as master regulators of adaptation of cancer stem/progenitor cells and their differentiated progenies to oxygen and nutrient deprivation by modulating their stem cell-like properties and metabolic and survival pathways and by activating tumour-associated stromal cells. Future investigations are, however, required to more precisely establish the molecular mechanisms at the basis of specific functions of HIF-1α and HIF-2α, common and unique gene patterns modulated through these transcription factors and their cooperative interactions with other growth factors in various human cancers during disease progression under normoxic and hypoxic conditions.

The results from some pre-clinical studies have also underlined great importance of targeting HIFs and altered energy metabolism in cancer- and metastasis-initiating cells and their progenies as well as their supporting host cells to overcome the treatment resistance and thereby prevent disease relapse and the death of cancer patients. Data from clinical trials have revealed that some anti-angiogenic drugs may reduce tumour tissue oxygenation and consequently promote the aggressive behaviour of cancer cells and treatment resistance. Therefore, the targeting of HIFs represents an attractive adjuvant cancer therapy to simultaneously eradicate cancer cells and induce anti-angiogenic effects in highly hypoxic tumours.

We can now envision the possibility of performing expression analyses of distinct molecular biomarkers associated with hypoxia and altered metabolic pathways in addition to the current diagnostic tests to select cancer patients who are likely to respond to cancer therapies targeting hypoxia including inhibitors of HIF signalling network, glycolysis, lipogenesis and autophagy. These multi-targeted approaches should be more effective, alone or in combination with current anti-hormonal treatments, radiotherapy and/or chemotherapy, against aggressive, metastatic and hypoxic tumours to eradicate total mass of cancer cells and cure cancer patients.
